# Epidemiology of Epilepsy in Lubumbashi, Democratic Republic of Congo

**DOI:** 10.1155/2020/5621461

**Published:** 2020-01-29

**Authors:** Olivier Mukuku, Pascal Nawej, Marcellin Bugeme, Frank Nduu, Paul Makan Mawaw, Oscar Numbi Luboya

**Affiliations:** ^1^Institut Supérieur des Techniques Médicales de Lubumbashi, Lubumbashi, Democratic Republic of the Congo; ^2^Centre Médical du Centre-Ville, Lubumbashi, Democratic Republic of the Congo; ^3^Faculté de Médecine, Université de Lubumbashi, Lubumbashi, Democratic Republic of the Congo

## Abstract

**Background:**

Epilepsy is one of the most common neurological conditions, but the majority of epilepsy patients in sub-Saharan countries do not receive appropriate treatment. In the Democratic Republic of Congo (DRC), particularly in Lubumbashi, very few epidemiological studies on epilepsy have emerged. This study aims to analyze demographic characteristics, semiology of epileptic seizures, and their etiologies in patients followed in hospital.

**Methods:**

This is a prospective descriptive study that enrolled 177 epileptic patients who performed a neurological consultation at the Centre Médical du Centre Ville (CMDC) in Lubumbashi (DRC) from January 1, 2016, to December 31, 2017.

**Results:**

The mean age of the patients was 20.0 years (range: 5 months and 86 years). The male sex was predominant (57.1%). The mean age at the seizure onset was 13.1 years, and the mean duration between onset of seizures and consultation was 83.5 months. The family history of epilepsy was present in 27.7%. Generalized tonic-clonic seizures were the most frequent (58.2%), followed by atonic generalized seizures (9.6%) and focal clonic seizures (8.5%). The etiology was found in 68 (38.4%) patients and was dominated by neurocysticercosis (26.5%), meningitis (25%), perinatal pathologies (20.6%), and head injury (20.6%).

**Conclusion:**

This study is a useful starting point from which health programs and health professionals can work to improve the diagnosis and quality of epilepsy management in our community.

## 1. Background

Epilepsy is a chronic condition, characterized by the repetition of paroxysms due to sudden, simultaneous, and abnormally intense activation of a large number of cerebral neurons [1]. Epilepsy is clinically characterized by sudden attacks that can be focal, when they involve only a part of the brain, or generalized, during which both hemispheres are involved. Epilepsy is one of the most common neurological conditions that can be experienced at any age and is a global public health problem. Globally, according to the recent World Health Organization (WHO) estimates, epilepsy is diagnosed in 2.4 million people each year, and nearly 80% of the 50 million people currently living with epilepsy are in low- and middle-income countries [2, 3]. It remains a serious disease in Africa due to late diagnosis and often inadequate management. The majority of epileptic patients in sub-Saharan countries do not receive appropriate treatment. In high-income countries, the annual incidence of epilepsy is between 30 and 50 per 100,000 people. In low- and middle-income countries, these numbers can be up to 2 times higher [3]. Its prevalence in sub-Saharan Africa is estimated between 5.2 and 74‰ [4]. Prevalences can be very variable from one region to another within the same country. These disparities in the prevalence of epilepsy can be explained by the study setting (urban or rural), by the existence of genetic factors predisposing to epilepsy in certain populations, and also by the type of investigations carried out (hospital surveys or door-to-door survey in the general population).

In the Democratic Republic of Congo, where very few epidemiological studies on epilepsy have emerged, it is estimated that more than 800,000 people suffer from epilepsy [5].

The objective of this study was to study the profile of epileptic patients followed at the Centre Médical du Centre Ville (CMDC) of Lubumbashi (Democratic Republic of Congo (DRC)) by describing the demographic characteristics, the semiology of epileptic seizures, and their etiologies.

## 2. Methods

This prospective descriptive transversal study conducted at the Centre Médical du Centre Ville (CMDC) of Lubumbashi (DRC) from January 1, 2016, to December 31, 2017. It involved 177 cases of epileptic patients who first performed a consultation for epilepsy in our hospital during this period.

We prospectively evaluated patients selected from our cohort consisting of 177 patients with epilepsy, who met the following inclusion criteria at the time of diagnosis:

- At least two unprovoked (or reflex) seizures occurring >24 h apart;
- One unprovoked (or reflex) seizure and a probability of further seizures similar to the general recurrence risk (at least 60%) after two unprovoked seizures, occurring over the next 10 years.

The seizures types were defined based on detailed analysis of the interview conducted by the main author. We asked the family members to film patients with a smartphone and bring us the videos. But before that we project to the patients and their family members some videos following the description of the seizures in connection with the patients. These videos are available on https://www.chusj.org/fr/soins-services/E/Epilepsie.

Electroencephalogram (EEG) standard (*n* = 116) and brain imaging (*n* = 91) were performed when necessary. No ictal EEG or video-EEG was registered. Patients with acute symptomatic seizures, an acute electrolyte imbalance, or paroxysmal nonepileptic events were excluded.

The data collection was done using a form gathering a number of items: age, sex, age at seizure onset, mean duration between onset of seizures and consultation, family history of epilepsy, etiology, and seizure type.

In order to define coherent entities, a grouping of the various epileptic manifestations was carried out on the basis of the last official classification of the different forms of epileptic seizures elaborated by the ILAE Commission for Classification and Terminology [6].

The clinical diagnosis of epilepsy was made on the basis of the operational definition of epilepsy of Fisher et al. [7]. The diagnosis of neurocysticercosis (NCC) was retained according to the diagnostic criteria proposed by Del Brutto et al. [8].

The data analysis was performed on the STATA 12 software. Descriptive statistic is presented as mean ± standard deviation.

## 3. Results

The age of patients was between 5 months and 86 years with a mean age of 20.0 ± 15.1 years. 92 (51.6%) patients were between 10 and 29 years old. The male patients were 101 out of 177 (M/F = 1.33) (Table 1).

**Figure 1:**
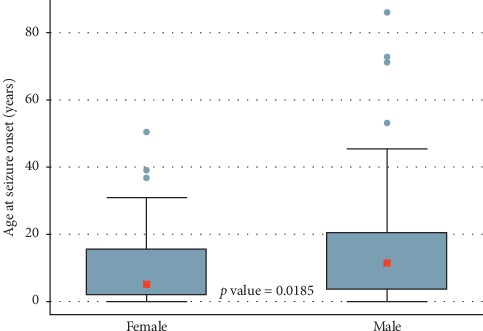
Age at seizure onset by sex.

The mean age at seizure onset was 13.1 ± 11.2 years (range: 5 days and 85.5 years). This mean age in men (15.4 years) was statistically higher than female patients (10.2 years; *p*=0.0185) (Figure 1).

The mean duration between onset of seizures and consultation was 83.5 months (range: 10 days and 440 months). Forty-eight (27.68%) patients had a family history of epilepsy.

Etiology was found only in 68 (38.4%) patients.

The main etiologies were confirmed or probable neurocysticercosis (26.5%), meningitis (25%), head injury (20.6%), and perinatal pathologies (20.6%) (Table 1).

**Figure 2:**
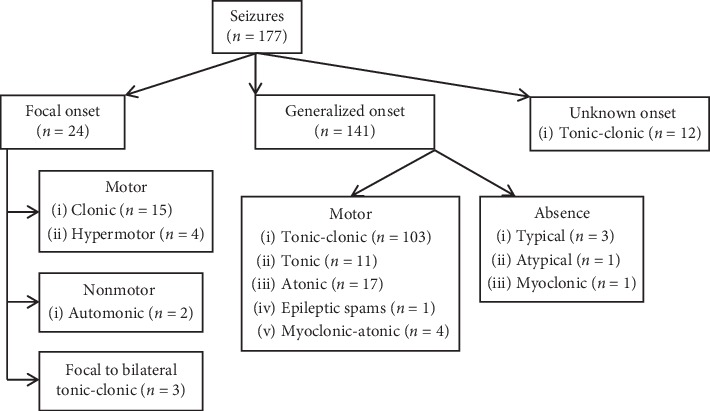
Expanded operational classification of seizure types in our cohort.

Generalized tonic-clonic seizures were the most frequent (58.2%), followed by atonic generalized seizures (9.6%) and focal clonic seizures (8.5%) (Figure 2).

## 4. Discussion

Epilepsy is a common neurological condition that has a devastating effect on people with it and even their families.

Some populations believe that epilepsy is contagious, and the organic origin of the disease is not often recognized. Others think that there is a connection between the occurrence of crises and the full moon. Mistaken beliefs about epilepsy vary from one country to another and from one community to another. Given the supposedly mystical origins of epilepsy, patients and their families prefer traditional medicine rather than modern medicine insinuating that the latter is powerless against epilepsy. It is after failure of traditional medicine that patients and their families turn to modern medicine or sometimes combine the two kinds of medicine without informing doctors. The mean age at seizures onset was 13.1 years. In sub-Saharan Africa, seizures occurred before the age of 20 in more than 60% of cases [9].

This study shows a great delay between the onset of seizures and neurological consultation (mean 83.5 months). In Africa, the epileptic is still too often stigmatized because of the ignorance of the disease and supernatural or mystical beliefs. Epilepsy is often considered to be of mystical origin, and the people who suffer from it are often brought to traditional healers in the first place. Given the supposedly mystical origins of epilepsy, patients may want to preferentially address traditional healers rather than conventional care structures.

We noted a male predominance (sex ratio M/F of 1.33). According to Ngoungou et al. [4], this strong male representation could be explained by the social impact of epilepsy and single girls tending to hide their epilepsy not to lose the chance to get married because epilepsy is considered a handicap. In other previous studies, however, the authors found a female predominance [10, 11].

In our study, generalized tonic-clonic seizures were the most frequent (58.2%). Studies in sub-Saharan Africa have shown a predominance of generalized tonic-clonic seizures (around 60%) [9]. There is a good identification of generalized tonic-clonic seizures by the entourage of patients. These seizures are indeed the most spectacular. Absences, myoclonic seizures, and other types of generalized seizures have also been reported (Figure 2). Idiopathic generalized epilepsies account for nearly a third of all epilepsies and are genetically determined and affect otherwise healthy people of both sexes and all races. They may start in infancy, childhood, or adolescence, but some have an onset in adulthood [12].

On the contrary, there is an underestimation of the number of focal to bilateral tonic-clonic seizures, the focal onset of which is difficult to recognize by the clinic alone.

According to Newton and Garcia, the burden of epilepsy in developing countries is more than double that of high-income countries, probably because the incidence of risk factors is higher [13]. Clinicians in developing countries usually have only the simplest complementary examination, and the etiological diagnosis is based mainly on the interview and the clinic [4]. In Africa, many parasitic infections are responsible for epileptic seizures or sequelae epilepsies, and neurocysticercosis (NCC) appears to be the most common parasitosis of the central nervous system [9, 14]. It is estimated that NCC is responsible for a third of epilepsies in parts of South America [15]. In Africa, the prevalence of cysticercosis in epileptic patients varies on this continent between 5 and 50% [4, 9]. A Tanzanian study also found that NCC is a major cause of epilepsy [16]. In the DRC, it is poorly known because of insufficient or nonexistent diagnostic means and only few epidemiological surveys are carried out. Clinical cases have however been published [17, 18].

Among the identified etiologies found, meningitis was reported in 25% of cases in our study. Meningitis and bacterial encephalitis frequently result in epileptic seizures, including meningococcal meningitis. Mbonda et al. [19, 20] found 18% of sequelae epilepsies in 144 children hospitalized for bacterial meningitis in Yaoundé (Cameroon). As for head injury, it was noted in 20.6% of our patients in whom the etiological factor was found. According to Sander and Shorvon [21], around 8% of epileptic patients have a history of head injury; this rate is estimated at 5% in developed countries. The risk of developing epilepsy was 13 times higher after head injury as reported in a Nigerian study by Ogunniyi et al. [22]. These head injuries are often secondary to road traffic accidents, which are due to the lack of safety measures (lack of traffic control, absence of seatbelt, or helmet for motorcyclists, etc.) [23]. Sometimes they result from work or home accidents or violent sports [4].

Perinatal pathologies were found in 14 (7.9%) of 177 patients in our series. According to Ngoungou et al. [4], the proportions of probable perinatal causes of epilepsy are very variable in the literature ranging from 1 to 36% (around a mean of 9%). These causes are probably major in developing countries with high rates of unattended pregnancies, of deliveries without qualified assistance, of obstructed labor in underequipped environments, even home births that are frequently responsible for obstetric trauma, anoxia, or cerebral vascular ischemia [4, 24, 25]. In addition to these are neonatal infections, prematurity, and metabolic disorders that are sometimes poorly or unhealthily (due to underqualification of the nursing staff or lack of equipment) leading to an increased risk of neurological sequelae, disability, and epileptic seizures in surviving newborns.

## 5. Conclusion

The clinical profile of epileptic manifestations in this hospital population is no different from that reported in other developing countries. Early diagnosis and treatment of parasitic infestations are essential for the proper management of patients. This study also highlights the need to manage perinatal and infantile pathologies correctly. Extensive studies are useful to better understand the epidemiology of epilepsy in the city of Lubumbashi.

## Figures and Tables

**Table 1:** Distribution of patients by age, sex, and family history of epilepsy and etiologic factor.

Variable	Number (*n* = 177)	Percent
Age		
<5 years	21	11.8
5–9 years	29	16.2
10–19 years	53	29.7
20–29 years	39	21.9
30–39 years	15	8.4
≥40 years	20	11.2
Sex		
Male	101	57.1
Female	76	42.9
Family history of epilepsy		
Absent	128	72.3
Present	49	27.7
Etiologic factor		
Not found	109	61.6
Neurocysticercosis	18	10.2
Meningitis	17	9.6
Perinatal causes	14	7.9
Head injury	14	7.9
Sickle cell disease	2	1.1
Stroke	1	0.6
Alcoholism	1	0.6
Meningioma	1	0.6

## Data Availability

The datasheet used to support the findings of this study is available from the corresponding author upon request.

## Abbreviations

DRC: Democratic Republic of Congo
ILAE: International League Against Epilepsy
NCC: Neurocysticercosis
WHO: World Health Organization.
